# To tPA or Not to tPA: Two Medical-Legal Misadventures of Diagnosing a Cerebrovascular Accident as a Stroke Mimic

**DOI:** 10.5811/cpcem.2019.4.42186

**Published:** 2019-07-08

**Authors:** Malia J. Moore, Jonathan Stuart, Alexandra Humphreys, James A. Pfaff

**Affiliations:** * Carl R. Darnall Army Medical Center, Department of Emergency Medicine, Fort Hood, Texas; † Madigan Army Medical Center, Department of Emergency Medicine, Tacoma, Washington; ‡ Wharton Aldhizer & Weaver, Harrisonburg, Virginia; § San Antonio Military Medical Center, Department of Emergency Medicine, San Antonio, Texas

## Abstract

We present two recent successfully litigated malpractice cases in which patients with cerebrovascular accidents were misdiagnosed as stroke mimics. The first was diagnosed as a hemiplegic migraine, which occurs in only 0.01% of the population. The second was diagnosed as a conversion disorder, which ultimately has a neurologic etiology in 4% of cases. In both cases, issues of poor patient communication and poor documentation were paramount in the legal outcome. We discuss caveats of stroke mimics, tissue plasminogen activator administration liability, and pitfalls in patient and family interactions.

## CASE REPORT

### Case 1: Estate of Smith versus Baca, Turner, and Augusta Emergency Physicians – Virginia^1^

A 40-year-old female was visiting her family when she had sudden onset of severe headache, with associated slurred speech, right-sided weakness, dizziness, nausea and vomiting. Her mother called emergency medical services (EMS) within five minutes and told the dispatcher she was concerned her daughter was having a stroke. EMS arrived at the home 40 minutes later, and the patient arrived at the hospital two hours and twenty minutes after onset of her symptoms. Transport was lengthy because the patient lived in a remote location. Although EMS initially considered flying her from the scene, they were forced to use ground transport due to poor weather; however, the paramedic on scene stated she complained only of headache and right-sided tingling. He performed a Cincinnati Prehospital Stroke Scale nine times during transfer, all of which were documented as normal.

On emergency department (ED) arrival, the patient was accepted from EMS by a nurse and the supervising emergency physician (EP), and was evaluated shortly afterward. Prochlorperazine, diphenhydramine, and dexamethasone were ordered to treat the patient’s headache, and a non-contrast computed tomography (CT) of the head was obtained. The EP diagnosed the patient with a complex migraine headache. It was documented but later disputed that her headache improved; however, while in the ED her symptoms changed. Her paresthesia moved to the left side, and five hours and ten minutes after onset of symptoms, a neurologist was consulted by phone and recommended magnetic resonance imaging (MRI), with a plan to see the patient in the hospital the next day. The MRI revealed an ischemic cerebrovascular accident (CVA). The patient died of complications three days later.

The plaintiff alleged that the EP should have initiated a stroke alert and consulted a neurologist immediately on arrival due to the patient’s history of symptoms at home, which could have been consistent with CVA. The hospital is a primary stroke center and had a tele-neurologist available. It was asserted that if the CVA had been identified and tissue plasminogen activator (tPA) administered within the accepted 4.5-hour window, Ms. Smith would have survived. The plaintiff also claimed that failure to identify and treat CVA was a departure from standard of care. Another EP who participated briefly the next morning and the neurologist were dropped from the case, as they were only involved after the tPA window had expired.

Furthermore, the family of the decedent asserted that they were concerned for stroke and requested multiple times that the patient be evaluated for stroke. Her mother was quoted in testimony as saying, “I want [the EP] to give her ‘that shot’” during the patient’s ED stay.

The EP and his defense team asserted that the decedent had a normal neurologic exam, with the exception of headache and arm tingling, which is not consistent with CVA. Her neurologic symptoms resolved rapidly and were associated with headache, which is consistent with the diagnosis of complex migraine. Furthermore, they alleged that even if CVA were thought to be the cause, the decedent would not have qualified for treatment with tPA based on accepted inclusion criteria. In fact, the experts testified that tPA is a potentially dangerous drug that has no scientific evidence of decreasing mortality; however, at least one jury member stated that the discussion of several, well-publicized tPA studies by the defense had the opposite intended effect, leading the juror to conclude that tPA would have helped the patient.

**CPC-EM Capsule**

What do we already know about this clinical entity?

Conversion disorder and hemiplegic migraine are diagnoses of exclusion after cerebrovascular accident has been ruled out.

What is the major learning point?

Tissue plasminogen activator (tPA) malpractice cases more often arise from not using it for stroke mimics, rather than complications from using it. Documenting is key.

How might this improve emergency medicine practice?

Litigation is less likely when there has been full consideration of diagnoses, and discussion with patients and families of the risks and benefits of administering tPA.

A major feature of the trial was discussion of the EP’s documentation. Several points were examined. A review of systems was documented, but the EP was unable to recall at trial which questions he would have included. A National Institutes of Health Stroke Scale (NIHSS) was never documented, despite the hospital being a primary stroke center. The physician documented only that the patient had “normal movement and coordination,” but the family disputed that he had ever examined the patient. Similarly, when the patient’s symptoms changed, the EP documented “normal strength and sensation;” however, the family stated there was also slurred speech, which was neither confirmed nor denied by the EP’s chart.

Most importantly, the initial chart presented in discovery for the trial was later found to have been altered by the EP once he learned the patient’s MRI results. The chart in question was an electronic medical record, in which the EP later overwrote his original chart to include the differential of CVA and state why he thought the presentation was more likely consistent with complex migraine. He did not address the potential use of tPA in either chart. He did not annotate that this was a change to the record being entered after the fact, when he had more information.

The family specifically testified that they felt the EP was dismissive, did not examine or care about the patient, and did not discuss options with them.

The jury returned a $3.5 million verdict for the plaintiff, which is currently under appeal.

### Case 2: Anonymous versus Anonymous – Kentucky

In a recently settled Kentucky case, a 43-year-old woman was celebrating on Christmas Eve with her family. There was no family discord or stress. She was witnessed to suddenly fall to the ground. The family noted that at approximately 9:30 pm she could not speak and was unable to use her right arm. They brought her immediately to the ED. Upon arrival, she was evaluated by the EP at 10 pm. A CT of the brain was performed and read by the radiologist as normal at 10:48 pm. Nursing notes commented on the patient’s “unwillingness” to communicate and also that she moved her extremities when the family was not present. The EP failed to document a neurologic exam on the chart. The family repeatedly implored the EP to be concerned about the patient’s condition. The family testified that the EP stated, “Your daughter is having a nervous breakdown because of how you raised her.” The patient was admitted to the hospital with a diagnosis of conversion disorder. No neurologist was consulted, and administration of tPA was not considered. The next morning, the patient was seen by the admitting physician, who suspected that she had suffered a CVA. A repeat CT revealed a large, left middle cerebral artery infarct. The patient was immediately transferred to a tertiary care facility but unfortunately was left with dense motor and speech deficits. She was unable to return to work and the burden of her care led to divorce. The case was settled for an undisclosed sum.^2^

## DISCUSSION

Dr. Moore: The time-sensitive administration of tPA is a “hot button” issue for many EPs. Controversy exists over the efficacy of the treatment, while providing treatment may directly cause acute decompensation and poor outcomes. Furthermore, several well-known societies support the use of tPA, including the American Heart Association and American Stroke Association, which have deemed its administration “definitely recommended.”^3^ Subsequently, several societies including the American Academy of Emergency Medicine have decried this claim.^4^ This leaves EPs in a position of fearing complications and medicolegal litigation whether from action or inaction.

In one retrospective cohort of 61,698 patients with acute ischemic stroke who presented within two hours of symptom onset, 25% of eligible patients failed to receive tPA treatment.^5^ The reason for this finding is likely multifactorial and thought to be a combination of patient factors such as dementia or other underlying comorbid disorders, hospital factors such as ability to quickly rule out hemorrhagic stroke and initiate therapy with tPA, and lastly EP decision-making. “By far the most common reason cited, reported in more than half of the patients, was mild or rapidly improving symptoms. Among patients without a documented reason for not receiving tPA, mild stroke symptoms, defined as a NIHSS score less than five, were strongly associated with a lower likelihood of being treated. Prior studies have found that this is the most common reason given for not treating otherwise eligible patients with intravenous (IV) tPA, yet multiple cohorts have found that up to one-third of patients with mild stroke symptoms at presentation will have poor long-term outcomes.”^5^

It can be easily extrapolated that EPs fear causing harm to a patient with mild symptoms; however, the risks associated with tPA are primarily applicable to patients who have already had an insult to brain tissue. In a study reviewing the effects of tPA in stroke mimics, 14% of their 521 patient cohort were ultimately diagnosed as stroke mimics after receiving tPA within three hours of symptom onset. None of these patients experienced intracranial hemorrhage. The most common stroke mimics were seizure, complex migraine, and conversion disorder.^6^

In addition to causing harm to a patient, EPs dread the outcome of litigation. In one study reviewing verdicts involving tPA, 33 cases were reviewed. The majority (N21; 63.6%) decided in favor of the defendant providers. Of the remaining, nine (27.3%) resulted in plaintiff verdicts, two (6.1%) resulted in settlements, and one (3.0%) was an arbitration favorable to the plaintiff. Compensation for plaintiffs ranged from $100,000 to $30 million. The most common claim plaintiffs made was a failure of the treating physician to provide tPA (N29; 87.9%), with only three cases (9.1%) in which patients sued the providers and claimed that the use of tPA caused their injury. In general, plaintiffs also claimed failure or delay in stroke diagnosis (N22; 66.7%). Based on this review, it appears that most successful litigation resulted from EP delay in stroke diagnosis and/or failure to administer tPA.^7^

A subsequent review confirmed these findings, where out of 40 applicable cases with available verdicts, the most frequent plaintiff claim was related to failure to administer IV tPA (38, 95%), and only two (5.0%) claims involved complications of treatment with tPA.^8^

As with many studies reviewing medicolegal verdicts, juries typically find in favor of the defendant physician, and this trend is also demonstrated with regard to tPA verdicts. Yet it appears that the number of cases is increasing. During the five years after tPA was approved by the United States Food and Drug Administration (1996 to 2001), there were only five cases, whereas from years six to ten (2002 to 2007), 28 cases were reported.^7^

Another issue that becomes critical in regard to administration of tPA is informed consent. A possible explanation for the preponderance of plaintiffs’ success with “failure to treat” cases is that most often when patients receive tPA they have also given informed consent, whereas when tPA was withheld, patients have often not received the same guidance and opportunity to choose. In these cases, either the stroke was not identified, or the physician unilaterally chose for other reasons (contraindications, patient comorbidities, personal concerns about efficacy) not to administer tPA. In other words, informed consent is critical, and in cases where tPA was not given, the physician was more likely not to have obtained or documented informed consent regarding the decision.

“Regardless of one’s personal view regarding the efficacy and safety of tPA, it is essential to discuss and document with patient and family (surrogate) all treatment options. Maintaining legible, detailed and timely documentation as to time of onset, examination findings and informed consent why patients should or should not receive tPA should substantially reduce the threat of legal action.”^9^

Despite the medical concerns involved in the case, lessons apparent for EPs are to listen to the patient and his or her family, validate and discuss their concerns, ensure they are provided informed consent and that they understand the major decision points in the patient’s care. Subsequently, all care should be documented completely and accurately. Occasionally, the situation may occur where the EP knows more at the time of charting than he or she did at the time of the patient’s care, and it is the opinion of the authors that this is best approached with transparency. For example, a statement such as, “I am addending the chart and am at this point aware the patient had X outcome. I want to record additional thoughts, exam, and findings performed and considered, but not fully documented at the time of care,” may be an appropriate introduction.

Dr. Stuart: Conversion disorder is characterized by neurologic symptoms causing distress but inconsistent with a specific medical or neurologic disease process. The diagnosis requires the following: altered motor or sensory function; clinical findings demonstrating incompatibility between the symptom and recognized neurological and medical conditions; an explanation for the symptom that is not better explained by another medical or mental disorder; and, finally, the condition must cause clinically significant distress or impairment.^10^ There is no requirement to identify psychological factors associated with the symptoms.

A 2005 review of 27 studies found 4% of patients diagnosed were subsequently found to have a neurologic disorder.^11^ More recent studies demonstrate a lower incidence of misdiagnosis. Nonetheless, a diagnosis of conversion disorder introduces etiologic assumptions that often cannot be proven and should be made with extreme caution, if at all, in the ED.^12^ Patients may also be frightened by the effects of neurologic symptoms and exaggerate symptoms to convince a physician of their problem, creating a presentation suggestive of a conversion disorder. Patients with conversion disorder may also have an underlying neurologic disease similar to patients with psychogenic, non-epileptic seizures also having a concurrent seizure disorder in some cases.

Hemiplegic migraine describes a migraine headache accompanied by motor weakness during the aura phase. Motor weakness never occurs in isolation and is often accompanied by other forms of aura leading to impairment.^13^ Most patients with hemiplegic migraine have headaches of varying severity and some will have more severe attacks accompanied by encephalopathy or coma.^14^ It is a rare disorder with clinically indistinguishable familial and sporadic forms, which together occur in only 0.01% of the population.^15^ Motor and sensory findings more often involve the upper extremities, and upper motor findings such as a Babinski sign or unilateral hyper-reflexia may be present during attacks.^16^

Attacks typically last hours followed by normalization of the neurologic exam, but some patients with familial forms may also have cerebellar findings between attacks.^14^ The diagnosis is clinical, and the occurrence of episodic, reversible motor weakness as a manifestation of migraine aura in conjunction with at least one other kind of aura is required. This diagnosis of a rare disease with features indistinguishable on acute presentation from a CVA is further complicated because up to 27% of stroke patients have headache at onset.^17^

Several additional stroke mimics exist and comprise 5–30% of patients initially diagnosed with stroke in the ED.^18^,^19^ Ictal and post-ictal deficits are the most common mimic (13–20%) followed by toxic and metabolic disorders (18%, primarily hypoglycemia), syncope (8–15%), and sepsis (10%).^19^ Vertigo and nystagmus, although common neurologic symptoms encountered in the ED, are caused by a stroke 3–10% of the time. Numerous additional stroke mimics exist with varying frequencies including, but not limited to, encephalopathies, mitochondrial disorders, multiple sclerosis, intracranial hemorrhage, reversible constriction syndromes, and transient global amnesia.^20^

Dr. Pfaff: Multiple studies have defined reasons that patients will initiate a malpractice action. One survey of 227 patients who were initiating claims for medical malpractice noted the actions were not only for the injury but also because of poor communication and insensitive handling. Only 15% of explanations were deemed satisfactory. The four main reasons given for litigation were the following: 1) Concerns about competence and prevention of other patients being harmed; 2) to obtain an understanding and explanation of what happened; 3) to obtain compensation to handle further medical care and permanent injury; and 4) to hold parties accountable for their actions.^21^ Another representative study linked the relationship of patient satisfaction to the likelihood of litigation. It reported on 353 physicians who had patient satisfaction surveys and ranked them into thirds. The middle third had a 26% higher chance of a malpractice action, while the lowest third had 110% higher risk.^22^

The importance of documentation can’t be emphasized enough in order to prevent and defend a malpractice action. Prosecuting attorneys use charting deficiencies to show carelessness, sloppiness, and dishonesty. Physicians should realize they themselves have the ability to control and create the evidence and facts with proper documentation.^23^

In a recent illustrative case of CVA in Louisiana, in which a physician was accused of failing to give tPA, the jury stated it did not feel tPA was indicated. The physician claimed he discussed the issue with the patient’s wife, but there was no discussion documented on the chart. The jury in an interview later stated the $300,000 award was solely based on the lack of documentation of informed decision-making.^24^

Humphreys JD: The axiom “if it’s not charted, it’s not done” rings especially true in medical malpractice litigation, where the patient’s medical records are often the only way for a physician to prove that a patient’s treatment was carried out properly. A physician’s failure to document the completion of certain tests or assessments can serve as persuasive evidence in showing the physician failed to perform a complete workup. Additionally, a physician’s alteration of records can also help a plaintiff’s case, especially if the changes were made after the physician learned or should have learned of an adverse patient outcome, by suggesting the physician’s acknowledgment of an earlier medical error. These general truisms came into play in both of these cases.

## CONCLUSION

When facing the possibility of a neurologic emergency in the ED, serious and debilitating possibilities should be considered, and rare conditions as well as those that can’t be confirmed with certainty should be less emphasized. It is imperative to both make a timely diagnosis and provide treatment for patients with a CVA. Physicians must also effectively communicate concerns and options to patients and their families (with documentation) to minimize subsequent litigation.
